# Physcion Mitigates LPS-Induced Neuroinflammation, Oxidative Stress, and Memory Impairments via TLR-4/NF-кB Signaling in Adult Mice

**DOI:** 10.3390/ph17091199

**Published:** 2024-09-11

**Authors:** Sareer Ahmad, Kyonghwan Choe, Haroon Badshah, Riaz Ahmad, Waqar Ali, Inayat Ur Rehman, Tae Ju Park, Jun Sung Park, Myeong Ok Kim

**Affiliations:** 1 Division of Life Sciences and Applied Life Science (BK 21 Four), College of Natural Science, Gyeongsang National University, Jinju 52828, Republic of Korea; sareerkhan2020@gnu.ac.kr (S.A.); k.choe@gnu.ac.kr (K.C.); hbadshah@awkum.edu.pk (H.B.); riazk0499@gnu.ac.kr (R.A.); waqarali_93@gnu.ac.kr (W.A.); inayaturrehman201516@gnu.ac.kr (I.U.R.); 2 Department of Psychiatry and Neuropsychology, School for Mental Health and Neuroscience (MHeNs), Maastricht University, 6229 ER Maastricht, The Netherlands; 3 Department of Pharmacy, Abdul Wali Khan University, Mardan 23200, KPK, Pakistan; 4 Haemato-Oncology/Systems Medicine Group, Paul O’Gorman Leukaemia Research Centre, Institute of Cancer Sciences, College of Medical, Veterinary & Life Sciences (MVLS), University of Glasgow, Glasgow G12 0ZD, UK; t.park.1@research.gla.ac.uk; 5 Alz-Dementia Korea Co., Jinju 52828, Republic of Korea

**Keywords:** Alzheimer’s disease (AD), neuroinflammation, oxidative stress, synapsis, lipopolysaccharide (LPS)

## Abstract

Alzheimer’s disease (AD) is the most predominant cause of dementia, considered a progressive decline in cognitive function that ultimately leads to death. AD has posed a substantial challenge in the records of medical science over the past century, representing a predominant etiology of dementia with a high prevalence rate. Neuroinflammation is a common characteristic of various central nervous system (CNS) pathologies like AD, primarily mediated by specialized brain immune and inflammatory cells, such as astrocytes and microglia. The present study aims to elucidate the potential mechanism of physcion that mitigates LPS-induced gliosis and assesses oxidative stress in mice. Physcion reduced the reactivity of Iba-1- and GFAP-positive cells and decreased the level of inflammatory cytokines like TNF-α and IL-1β. Physcion also reversed the effect of LPS-induced oxidative stress by upregulating the expression of Nrf2 and HO-1. Moreover, physcion treatment reversed LPS-induced synaptic disorder by increasing the level of presynaptic protein SNAP-23 and postsynaptic protein PSD-95. Our findings may provide a contemporary theoretical framework for clinical investigations aimed at examining the pathogenic mechanisms and therapeutic approaches for neuroinflammation and AD.

## 1. Introduction

Alzheimer’s disease (AD) has been a significant threat in medical care history for the past ten decades and is a major source of dementia. It has an immense occurrence rate, affecting almost 40 million people around the globe in terms of reported cases, and is projected to increase in upcoming years [1,2]. It is the most frequent neurodegenerative disease and is a partial aspect of the aberrant accumulation of beta-amyloid (Aβ) oligomers [3]. Besides amyloid plaques, neuroinflammation is a shared feature of different central nervous system (CNS) pathologies which is regulated mainly by specialized brain immune and inflammatory cells, including astrocytes and microglia [4,5,6]. Certain pathogens, such as viruses and bacterial infections, can have an effect on the CNS, whether in the brain or in the peripherals. Among these, acute systemic inflammatory processes caused by lipopolysaccharide (LPS), the most frequent antigen on the cell surface of Gram-negative bacteria, can lead to neuronal death and neurodegeneration [5,7], particularly by inducing neuroinflammation, which is one of the pathogenic causes of neurodegeneration and frequently occurs prior to the onset of neurodegenerative diseases like AD [8,9].

LPS, which is a neuroinflammation-inducing factor, is an immunostimulatory cell-wall component of Gram-negative bacteria, first identified as a ligand for Toll-like receptor 4 (TLR-4), which is primarily expressed on microglia [10,11,12]. Microglia and astrocytes are the most abundant and widely distributed cells in the CNS, and interact with neurons and immune cells [13]. Several investigations have demonstrated that LPS is responsible for activating microglia and astrocytes and has the potential to enhance the expression level of pro-inflammatory cytokines, which in turn induce interleukin IL-1β, tumor necrosis factor (TNF-α), and nitric oxide (NO), specifically through the activation of nuclear factor-κB (NF-κB) signaling [14], further, leading to oxidative stress and synaptic failure [13], which are defining characteristics of AD [5,15]. LPS also promotes the activation of innate immunity, causing mitochondrial dysfunction [16], leading to elevated reactive oxygen species (ROS) production causing antioxidant defense system disruption, and contributing to the early stages of AD [17]. The nuclear factor erythroid 2-related factor 2 (Nrf2) is a transcription factor and serves as a crucial regulator of the antioxidant defense system [18]. Nrf2 modulates the basal and stress-induced expression of numerous antioxidant response element (ARE)-dependent genes, thereby governing the physiological and pathophysiological outcomes of oxidative stress [19,20]. The balance between ROS levels and NRF2 activation is crucial in determining cell fate, influencing processes like cell survival, programmed cell death, cancer development, and aging. The proper regulation of the NRF2 pathway is essential for maintaining cellular health and protecting against damage caused by oxidative stress [21,22]. Elevated levels of oxidative stress and neuroinflammation disrupt the structure and function of neurons, impairing synaptic activity, memory, and cognitive functions in the brain [23].

There has been an increasing interest in investigating natural products as a potential treatment agent for neuroinflammation in recent years [24]. Many medicinal plants and their secondary metabolites have been reported to possess the ability to improve symptoms of neurodevelopmental disorders [25]. Physcion is a naturally occurring anthraquinone derivative found in medicinal plants (e.g., *Reynoutria japonica*, *Rheum tanguticum*, and *Reynoutria emodi*, etc.) as well as vegetables like cabbage, lettuce, and beans [26]. Physcion exhibits many pharmacological effects, including anti-inflammatory, anti-tumor, and anti-microbial characteristics [27]. Previous studies have shown that the oral administration of physcion exhibits minimal toxicity and is capable of crossing the blood–brain barrier [28]. Past studies of physcion related to neuroprotective effects have mostly focused on preventing brain damage caused by ischemia, hypoxia, and ischemia–reperfusion injury [6,29]. In addition, a previous study reported that physcion reduces ROS and NO levels, while significantly reversing the effects on NF-κB and TNF-α in a dose-dependent manner. Moreover, physcion protects HUVECs from PA-induced injury by inhibiting endoplasmic reticulum (ER) stress signaling. These results suggest that physcion may alleviate Palmitic acid-induced inflammation by inhibiting the NF-κB/TNF-α signaling pathway [30]. Currently, the effects of physcion on LPS-induced neuroinflammation have not yet been investigated. Therefore, the present study aims to evaluate the potential mechanism of physcion in lowering LPS-induced gliosis and elevated oxidative stress in mice. Our present findings may uncover the most recent theoretical foundation for clinical research, along with investigating the pathogenic mechanism and treatment of neuroinflammation, which can be a contributing factor in the development and optimization of various neurodegenerative disorders like AD.

## 2. Results

### 2.1. Physcion Treatment Prevents LPS-Induced TLR4 Signaling and Glial Cell Reactivity in the Mouse Brain

**Figure 1:**
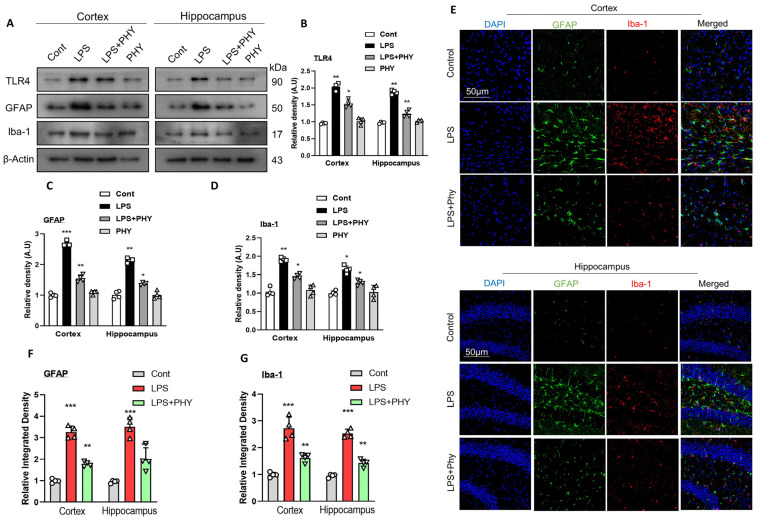
Physcion treatment prevents the LPS-induced activation of TLR4 and glial cells in the cortex and hippocampus of the mouse brain. (**A**–**D**) Images of the scanned Western blot results showing the expression of TLR4, GFAP, and Iba-1 proteins in the cortex and hippocampus of mouse brains after LPS and Physcion treatment, while bar graphs depict the difference. (**E**–**G**) Illustrative confocal images accompanied by a bar graph showing the relative integrated density of GFAP and Iba-1 in the mouse brain cortex and hippocampus (DG region). Magnification 10× and scale bar 50 µm. The data are presented as the mean ± standard error of the mean (*n* = 4 mice per group). The asterisk (*) sign indicates a significant difference among groups; significance  =  * *p*  <  0.05; ** *p*  <  0.01; *** *p*  <  0.001.

The crystal structure of the TLR4-MD-2-LPS complex has illuminated the role of TLR4 receptor signaling in the progression of LPS-induced inflammatory responses. Additionally, the structure–activity relationship of LPS has provided a clear understanding of how LPS interacts with TLR4 receptors [31]. Recent research suggests that TLR plays a significant role in mediating interactions between neurons and glial cells in the CNS [32]. Iba-1 and GFAP are widely recognized markers used to identify microglia and inflammatory astrocytes [33]. Western blot and immunofluorescence analysis were performed to investigate whether physcion could undo the LPS-induced relative protein abundance of TLR4, astrocytes, and microglia in mouse neuronal tissues. Our immunoblot results indicated that the relative protein abundance of TLR4, GFAP, and Iba-1 in the cortex and hippocampus of the LPS group was significantly higher than that of the saline-treated group. However, after the treatment of physcion (LPS + PHY), the relative protein density of TLR4, GFAB, and Iba-1 expression were significantly reduced compared to the LPS-alone group. (Figure 1A–D). To strengthen our immunoblot results, the immunofluorescence of GFAP was also examined. The immunofluorescence analysis revealed that the expression of GFAP in the LPS-treated group was significantly higher than that of the normal saline-treated group. Remarkably, when compared to the LPS-alone group, the LPS + PHY treatment substantially decreased the immunofluorescence reactivity. According to the immunoblot and immunofluorescence results, Physcion can decrease the relative protein abundance of TLR4 and reactivity of glial cells in the mouse brain. (Figure 1E,F).

### 2.2. Physcion Inhibits the Activation of NF-κB and Inflammatory Cytokines

Different studies revealed that LPS increases the level of pro-inflammatory cytokines [34,35].

**Figure 2:**
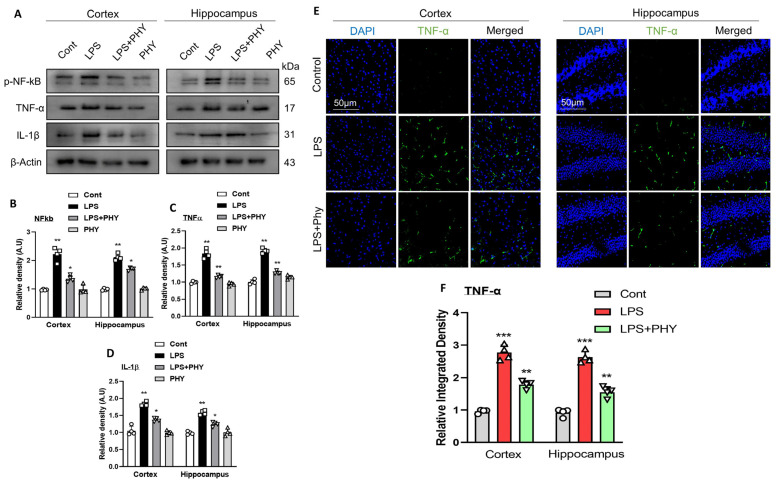
Physcion treatment prevents the LPS-induced activation of NF-κB, IL-1β, and TNF-α markers in the cortex and hippocampus of the mouse brain. (**A**–**D**) Images of the scanned immunoblot results showing the expression of NF-κB, IL-1β, and TNF-α proteins in the cortex and hippocampus of mouse brains after LPS and Physcion treatment, while bar graphs depict the difference. (**E**,**F**) Illustrative confocal images accompanied by a bar graph showing the relative integrated density of GFAP in the mouse brain cortex and hippocampus (DG region). Magnification 10× and scale bar 50 µm. The data are presented as the mean ± standard error of the mean (*n* = 4 mice per group). The asterisk (*) sign indicates a significant difference among groups; significance  =  * *p*  <  0.05; ** *p*  <  0.01; *** *p*  <  0.001.

NF-κB (nuclear factor kappa-light-chain-enhancer of activated B cells) is a key transcription factor that plays a pivotal role in regulating immune responses, inflammation, and cell survival. It becomes activated by numerous stimuli, such as stress and cytokines. NF-κB drives the expression of genes encoding inflammatory cytokines like IL-1β (interleukin-1 beta) and TNF-α (tumor necrosis factor-alpha), which are critical for the onset and progression of inflammatory processes [36]. Western blot and immunofluorescence were performed to assess the activation of the NF-κB and inflammatory cytokines such as TNF-α and IL-1β in the mouse brain. In comparison to the normal saline-treated groups, the LPS groups had higher levels of NF-κB, TNF-α, and IL-1β protein expression. Similarly, when the LPS-alone group was analyzed with the LPS + PHY group, there was a decrease in NF-κB, TNF-α, and IL-1β in both the cortex and hippocampus (Figure 2A–D). For further confirmation, the immunofluorescence detection of TNF-α was performed, which showed that Physcion effectively reduced the relative amount of protein TNF-α in the cortex and hippocampus of the mouse brain. (Figure 2E,F).

### 2.3. Physcion Reduced LPS-Induced Oxidative Stress in the Mouse Brain

**Figure 3:**
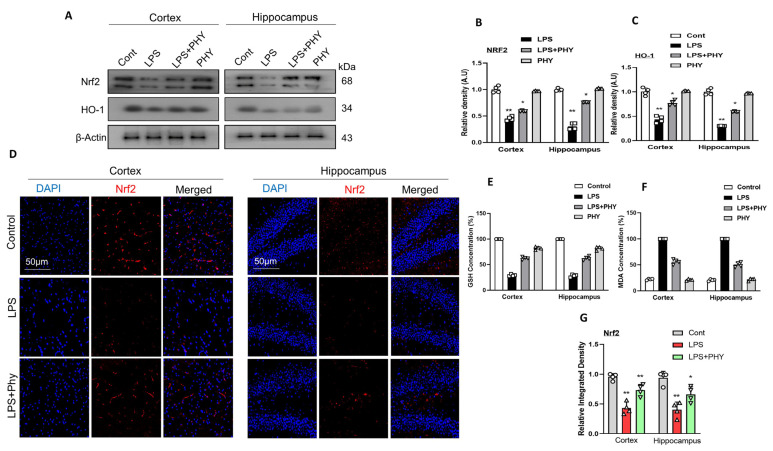
(**A**–**C**) Physcion treatment prevents LPS-induced oxidative stress in the mouse brain. Images of the scanned immunoblots results showing the expression HO-1 and Nrf2 in the cortex and hippocampus of mouse brains after LPS and Physcion treatment, while bar graphs depict the difference. (**D**,**G**) Illustrative confocal images accompanied by a bar graph showing the relative integrated density of Nrf2 in the mouse brain cortex and hippocampus (DG region). Magnification 10× and scale bar 50 µm. (**E**,**F**) GSH and MDA assays. The asterisk (*) sign indicates a significant difference among groups; significance  =  * *p*  <  0.05; ** *p*  <  0.01.

An imbalance in redox homeostasis leads to heightened oxidative stress, damage to biomolecules, compromised neuronal structure, and the death of neuronal cells, all of which play a role in the development of various neurodegenerative diseases [37]. We also assessed the anti-oxidative effect of Physcion against LPS-induced oxidative stress in the mouse cortex and hippocampus. We carried out a Western blot for Nrf2 and HO-1. According to our results, the protein expression of Nrf2 and HO-1 decreased in the cortex and hippocampus of the LPS-treated mouse brain. Interestingly, after the physcion administration, the LPS + PHY group protein expression was upregulated as compared to the LPS-alone group (Figure 3A–C). For further confirmation of antioxidant potential, we performed assays including GSH and LPO. Antioxidant enzyme concentrations (GSH) in cortical and hippocampal tissue were reduced after LPS treatment, but were significantly increased after receiving physcion. Similarly, when LPS was compared to the normal control group, there was a statistically significant rise in MDA (*p* < 0.001). Physcion’s attenuative potential was further supported by measuring the MDA level, which was considerably lower (*p* < 0.001), demonstrating physcion’s useful involvement in oxidative stress. To strengthen our Western blot results, we also performed immunofluorescence staining for Nrf2, which showed that the physcion dose significantly upregulated Nrf2 in the cortex and hippocampus (Figure 3D,E).

### 2.4. Physcion Treatment Improved LPS-Induced Synaptic Damage and Memory Impairments

**Figure 4:**
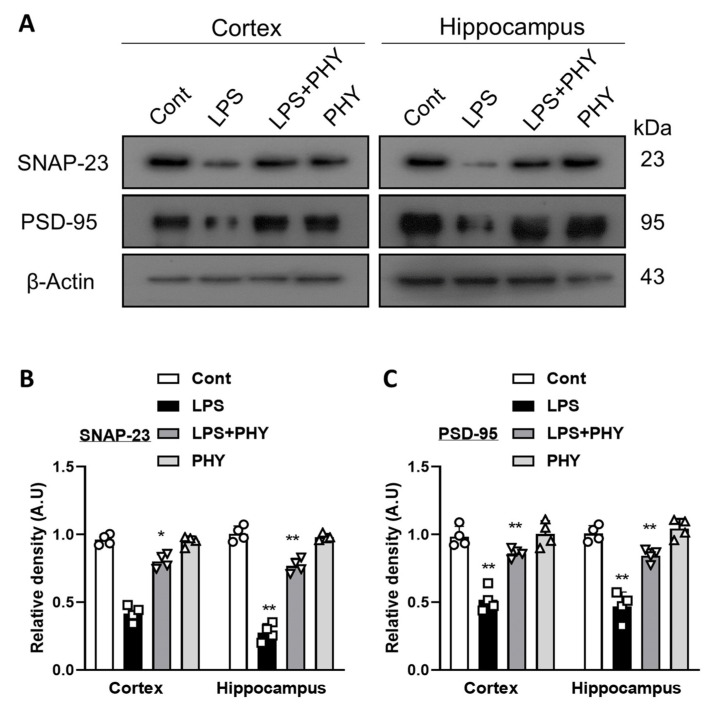
Physcion treatment prevents LPS-induced cognitive impairment. (**A**–**C**) Images of the scanned immunoblots results showing the expression of synaptic proteins PSD-95 and SNAP-23 in the cortex and hippocampus of mouse brains after LPS and Physcion treatment, while bar graphs depict the difference. Magnification 10× and scale bar 50 µm. The asterisk (*) sign indicates a significant difference among groups; significance  =  * *p*  <  0.05; ** *p*  <  0.01.

Different studies have reported that LPS causes memory impairment and synaptic dysfunction [5,38]. Neuroinflammation primarily leads to neuronal and synaptic dysfunction, which are closely linked to memory loss and cognitive decline. The inflammatory processes in the brain can disrupt normal neural communication and synaptic plasticity, ultimately impairing the brain’s ability to process, store, and retrieve information, thereby significantly contributing to cognitive deficits [39]. We performed Western blot analysis to examine the synaptic proteins, including postsynaptic density protein 95 (PSD-95) and synaptosomal-associated protein 23 (SNAP-23). The immunoblot result shows that the expression of PSD-95 and SNAP-23 was significantly decreased in the cortex and hippocampus of the LPS group as compared to the normal saline-treated group. However, after physcion treatment, the PSD-95 and SNAP-23 levels of protein expression were significantly increased compared to the LPS-alone group (Figure 4A–C). Our results suggest that physcion may improve the synaptic dysfunction of mouse brains.

### 2.5. Physcion Enhances Cognitive Behavior in Mice

**Figure 5:**
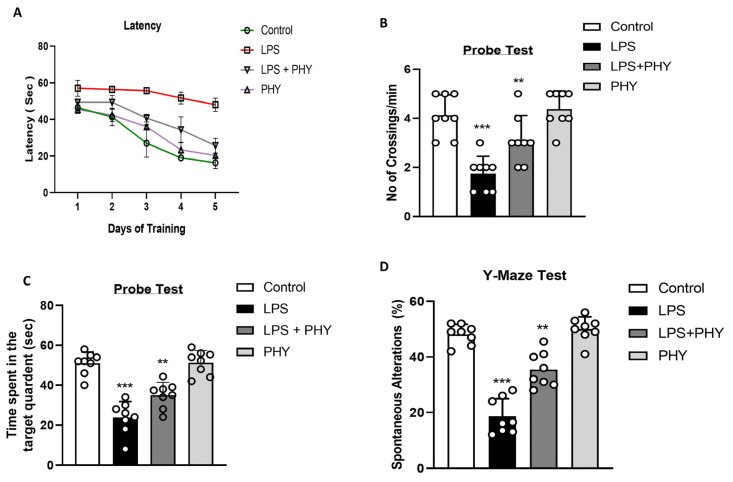
Physcion treatment prevents LPS-induced memory impairment and cognitive dysfunction (**A**) Line graph depicting the average escape latency over training days to reach the visible platform in the MWM task. (**B**) The number of crossings near the platform. (**C**) Time spent in the target quadrant. (**D**) Y-maze task for measuring the percentage of spontaneous alternation behavior in different groups. The results are shown as the mean ± SEM (*n* = 8 mice/group). The asterisk (*) sign indicates a significant difference among groups; significance  = ** *p*  <  0.01; *** *p*  <  0.001.

To determine the protective effect of physcion against LPS-induced cognitive and memory impairment, we performed behavioral experiments using the Morris water maze (MWM) and Y-maze. Eight mice in each experimental group were trained during MWM for five days to assess learning through practice using a hidden platform. According to our results, the escape latency of LPS-treated mice was significantly increased as compared to control mice, while physcion treatment significantly reduced escape latency and improved cognitive performance. Following the completion of the five-day training, we performed a probe test to identify the deficits in spatial learning. When the hidden platform was removed from the probe test, we observed a reduction in the number of crossings near the platform and reduced time spent in the target quadrant in LPS-treated mice. In contrast to LPS-treated mice, LPS + PHY-treated mice spent more time around the platform and in the target quadrant (Figure 5A–C).

To further examine the cognitive abilities of mice, we assessed spatial working memory, which is used to check short-term memory, using a Y-maze test for spontaneous alteration. The total number of arm entries, successive triplets, and exploratory movements were used to assess the percentage of spontaneous behavioral change. The results revealed that the percentage of spontaneous changes was lower in LPS-treated mice compared to the normal saline group. However, physcion administration significantly increased the percentage of spontaneous alteration behavior, suggesting that physcion treatment not only increases exploratory behavior but also reduces LPS-induced cognitive dysfunction (Figure 5D).

## 3. Discussion

The interplay between neuroinflammation and neurodegenerative disorders was previously regarded as an epiphenomenon, occurring when inflammation triggers an activation response in glial cells [40]. Various studies have reported that neuroinflammation plays a crucial role in cognitive impairment and neurological disorders [41]. In addition, accumulated evidence suggests that neuroinflammation-induced oxidative stress plays a more prominent role in neurodegenerative disorders. All these factors, including neuroinflammation in the form of reactive gliosis and reactive oxygen species, can lead to neuronal damage, resulting in cognitive impairment characteristic of Alzheimer’s disease [42]. Therefore, the current study was conducted to evaluate the effect of physcion against an entirely inflammatory LPS-induced animal model to investigate the crosstalk between neuroinflammation, oxidative stress, and cognitive impairment. The biochemical and behavioral changes generated as endpoints supported the development of therapeutic approaches for neurodegenerative disorders [43].

The exact pathology of neurodegenerative diseases is still unclear, and treatment currently relies only on symptomatic relief. Therefore, developing appropriate animal models is critical for studying neurodegenerative disorders and the cognitive deficits associated with neuroinflammation [41]. Several studies have reported that LPS induces inflammation by activating microglia and astrocytes [44,45], as microglia play an essential role in immunological defense and inflammatory responses in the CNS [46]. Following a brain injury, reactive gliosis and scarring are frequent pathological processes in which the glial cells remain in the damaged area of the brain and secrete inhibitory substances to stop the formation of new cell growth [33]. In the present study, Western blot and confocal laser microscopic results revealed an increase in the expression of TLR4, Iba-1, and GFAP in the LPS-injected group. Interestingly, this overexpression of biomarkers was decreased in the LPS + PHY co-treated mouse group, hence demonstrating that PHY significantly counteracted the negative effects of LPS on the glial cells in the cortexes and hippocampi of the LPS-injected group, as indicated by the relative density of GFAP and Iba-1. In addition, many studies have revealed that immune cells in the CNS become activated by LPS due to the presence of TLR4 receptors on their surface, which can lead to increased neuroinflammation [47]. Furthermore, the excess accumulation of pro-inflammatory cytokines stimulates TLR4 receptors on microglia and astrocytes, triggering cytotoxic consequences and activating pro-inflammatory signaling pathways downstream, and vice versa. Furthermore, these cytokines are produced in greater amounts when microglia are exposed to LPS, indicating that the pathological build-up via LPS is a major mediator that controls neuroinflammation [48]. Ultimately, these mechanisms lead to neuroinflammation, synaptic loss, and neuronal death [49]. Similarly, the TLR-4 receptor plays a crucial role in mediating several inflammatory pathways and leads to the activation of NF-κB via myeloid differentiation factor 88-(MyD88-) [50]. Upon the activation of NF-κB, a multitude of neurotoxic pro-inflammatory chemicals, such as cytokines like TNF-α and IL-1β, are released [47]. In the current study, the expression levels of p-NF-κB, IL-1β, and TNF-α were also assessed in all experimental mouse groups. The LPS-injected mouse group showed an upregulation of these inflammatory cytokines, which were decreased in the LPS + PHY-treated group. Our results are consistent with prior research in which physcion inhibited the IFN-β-induced inflammatory response in HAPI cells by reducing the production of TNF-α and IL-1β [24].

Furthermore, the Nrf2/HO-1 pathway is crucial in neurodegenerative disorders as it regulates redox homeostasis, DNA repair, and mitochondrial autophagy [51]. The transcription factor nuclear factor (erythroid-derived 2)-like 2 (Nrf2), a key regulator of the oxidative stress response, binds to a cis-acting element known as the antioxidant responsive element (ARE), thereby protecting cells from oxidative stress-induced neuronal cell death [52]. The key approach to achieve anti-oxidation was the (NRF2/HO-1) signaling pathway’s scavenging of reactive oxygen species (O^2−^ and H_2_O_2_). This pathway is a promising factor for reducing cognitive impairment in neurodegenerative diseases [53]. Previous reports have demonstrated that most neurodegenerative diseases are linked to oxidative stress. LPS is also associated with increased levels of oxidative stress and inflammation [54]. Notably, our Western blot and confocal laser microscopic results also confirmed a decrease in the expression of Nrf2 and HO-1 in the LPS-injected group, which is consistent with previous research [46]. Interestingly, this downregulated expression of biomarkers was increased in the LPS + PHY co-treated mouse group, hence proving the antioxidant potential of physcion. Our results are consistent with a previous study in which physcion decreased the oxidative stress induced by OGD/R in SH-SY5Y cells as well as having antioxidant activity against the depolarization of the mitochondrial membrane and the buildup of ROS in the cytoplasm [28].

Moreover, the neurotoxicity and neuroinflammation that are caused by LPS lead to cognitive deficits and synaptic dysfunction [55,56]. One study suggested that synaptic proteins are changed in neurodegenerative disorders and are essential for the exocytosis and endocytosis of different neurotransmitters [57]. Research suggests that two known synaptic proteins, synaptosomal-associated protein 23 (SNAP-23) and PSD-95, which are important for both synaptic plasticity and cognitive function, are associated with LPS-induced synaptic dysfunction [58]. According to our results, the LPS-injected mouse group reduced the levels of PSD-95 and SNAP-23 in the mouse cortex and hippocampus. However, physcion treatment significantly improved the LPS-induced decrease in memory-related synaptic markers via increasing the relative density of PSD-95 and SNAP-23 in the mouse cortex and hippocampus. Similarly, in the behavioral analysis, the memory cognitive function was assessed via Morris water maze (MWM) test, and observed that physcion administration significantly reduced the longer escape delay, more time spent in the target quadrant, and platform crossings during the probing test. Furthermore, in the Y-maze test, the spontaneous alteration percentage (%) was increased after physcion treatment. Taken together, our results suggest that physcion treatment improves behavior and memory by reducing LPS-induced synaptic protein loss and declines. Based on these results, we hypothesized that physcion treatment is effective against LPS-induced TLR4 signaling concerning synaptic dysfunction and memory impairment.

There are certain limits to our study, despite the fact that it addressed a wide range of topics and made significant advance in our knowledge of the pathological effects associated with LPS-induced neuroinflammation and neurodegeneration. One of the main limitations is the very small sample size, which highlights the need for a larger study to clarify physion’s role in LPS-induced neuroinflammation. Typically, LPS causes acute neuroinflammation rather than the persistent inflammation that is frequently seen in a number of neurodegenerative illnesses. Similarly, LPS induces inflammation throughout the body, and our study might not accurately replicate the localized neuroinflammatory processes that are indicative of specific neurological conditions. Furthermore, LPS-induced inflammation might vary between species, so findings from research on rodents may not necessarily translate to people.

## 4. Materials and Methods

### 4.1. Chemicals

Physcion (1,8-Dihydroxy-3-methoxy-6-methylanthraquinone, Emodin-3-methyl ether, PHY, catalog number SC-205805), LPS, which is the major component of the outer membrane of Gram-negative bacteria, and the primary antibodies were purchased from Santa Cruz Biotechnology, (Dallas, TX, USA). The primary antibodies used in the study are shown in Table 1.

### 4.2. Animal Handling

Male C57BL/6 N mice aged 8 weeks were acquired from Samtako Bio (Osan, Republic of Korea) and were kept in the animal house for 12/12 h cycles of light and dark at 23 °C and 60 ± 10% humidity. Necessary food and water were administered ad libitum. Mice were acclimatized for one week to a new environment. Mice were treated and then maintained as per the guidelines issued by the Institutional Animal Care and Use Committee (IACUC) of the Division of Applied Life Sciences, Gyeongsang National University. All mice were handled according to the approved protocol for experimental method guidelines (Approval ID: 125, Code GNU 200331-M0020).

### 4.3. Animal Grouping and Treatments

**Figure 6:**
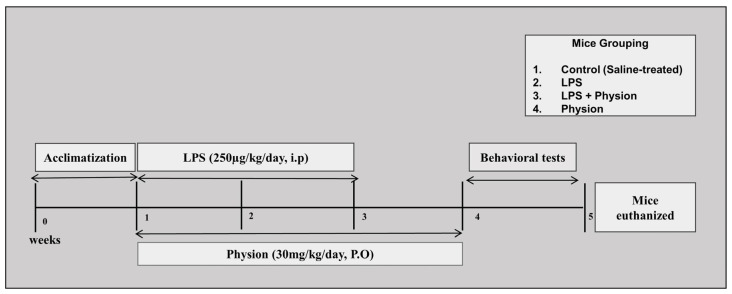
Shows the schematic representation of animal groupings and treatments.

All mice were divided into 4 groups after acclimatization of one week: (1) control mice administered with saline (I.P); (2) mice injected with LPS (250 µg/kg/day, I.P.) for two weeks (every alternative day); (3) mice treated with LPS + Physcion (30 mg/kg/day P.O) for 3 weeks (two weeks along with LPS injection and one week after the LPS injection); and (4) mice treated with physcion separately (30/mg/kg P.O), as shown in Figure 6. According to previous research, the reports showed that physcion treatment with a dose of 20 mg/kg and 40 mg/kg protects rat brain via inhibiting the TLR4/NF-κB pathway with similar significance [28], and hence we selected the average of these two doses for our current study. The stock solution was formed by dissolving physcion in dimethyl sulfoxide (DMSO). Fresh physcion solution was prepared every day in normal saline as per the required volume of injection (250 µL/kg/day). The same volume of dissolved LPS in saline was administered I.P to the mice. The mice were brought to the injection chamber daily at the same time to receive their shots.

### 4.4. Behavioral Study

A Morris water maze (MWM) test and a Y-maze test were conducted for a behavioral study (*n* = 8/group) to figure out the effect of physcion on memory function by using video-tracking software (SMART Panlab, Harvard Apparatus, Holliston, MA, USA).

MWM was carried out with a few changes to the previously published protocols [59]. Following five days of guidance, latency (s) was measured to determine how long it took to get to the hidden platform. Every day, behavioral analysis was carried out by the experiment plan, one hour after the drug was administered. After five days of training, on the following day, we conducted the probe test to assess memory consolidation by removing the platform and giving the mice a minute to swim around freely. Measurements were made of the number of crossings over the previously concealed platform and the amount of time spent in each target quadrant. Video-tracking software (SMART V3.0 Panlab Harvard Apparatus Bioscience Company, Holliston, MA, USA) was used to record the data.

The Y-maze test was also conducted to assess spontaneous alteration. The three arms of the transparent plastic Y-maze equipment were installed at a 120-degree angle to one another [60]. The length of each arm was 50 cm, the height was 20 cm, while the width was 10 cm both at the top and bottom. The Y maze test was performed after the completion of treatment for 5 days. Every mouse was given 8 min for three successive sessions and the starting point was the center of the apparatus. All the entries of mice were optically observed. Spontaneous alteration is defined as the mice’s successive entrance into the three arms in overlapping triplet sets. The calculation of finding (%) of alternation was as follows: [successive triplet sets (entries into three different arms consecutively)/total number of arms entries − 2] × 100.

### 4.5. Extraction of Protein from Mouse Brain

After the completion of the behavior study, all the mice were brought to the surgical room and anesthesia drugs were administered with 0.05 mL/100 g body weight Rompun (Xylazine) and 0.1 mL/100 g body weight Zoletil (ketamine). The mice were euthanized and killed by decapitation for Western blot analysis, while transcardial perfusion was performed for immunohistochemistry studies. Different groups of mice were used for each type of analysis to ensure proper sample preparation for the respective techniques. The cortex and hippocampus were separated immediately for further biochemical analysis and kept at −80 °C. As per the instructions given by the manufacturer (iNtRON Biotechnology, Inc., Dallas, TX, USA), brain tissue was homogenized by a PRO-PREPTM protein extraction solution. After that, the samples were centrifuged for 25 min at 4 °C and 13,000 rpm. Samples were collected and stored at −80 °C until further study.

### 4.6. Immunofluorescence Staining

Immunofluorescence staining was carried out as previously reported in the studies with few changes [61]. For morphological analysis, mice were anesthetized and perfused transcardially with ice-cold 1X PBS, followed by 4% neutral buffered paraformaldehyde (NBP). After 48 h of fixation in NBP, the brains were immersed in a 20% sucrose solution for 72 h and then placed in O.C.T. Compound. Coronal sections, each 14 μm thick, were obtained using a microtome (Leica, CM cryostat, Nussloch, Germany). The sections were then mounted on positively charged ProbOn slides (Thermo Fisher, Waltham, MA, USA) using a thaw technique in preparation for confocal microscopy. The slides that contained the brain tissues were washed with 0.01 M PBS for 15 min. Then, the blocking solution and proteinase K were added for six minutes, followed by washing two times with PBS. Primary antibodies (1:100) were added to the slides and kept at 4 °C overnight (Table 1). Secondary antibodies (FITC and TRITC conjugated, Santa Cruz Biotechnology, 1:50 in PBS) were added at room temperature after two washes with PBS and were incubated at room temperature for 90 min. After the secondary antibody, the slides were washed two times with PBS, and then DAPI was added for nucleus staining. Glass cover slips were put on the slides with a mounting medium. A confocal microscope (FluoView FV 1000 Olympus, Tokyo, Japan) was used to take the pictures.

### 4.7. Western Blot Analysis

Western blot was carried out with the previously described protocols to assess the concentration of different proteins associated with neuroinflammation in the cortex and hippocampus [62]. The amount of protein was measured in tissues of mouse brains using the Bio-Rad protein assay kit (Bio-Rad Laboratories, Hercules, CA, USA). An equal amount of protein samples was used in the gel electrophoresis, which adopted 10–12% BoltTM Mini Gels. Molecular weights were determined as a control by using a broad-range pre-stained protein ladder (GangNam-StainTM, iNtRON Biotechnology). Various gels which contained different protein bands were kept on the PVDF membranes. The PVDF membranes were placed for one hour in skim milk (5% *w/v* skim milk in 1X TBST) to reduce the non-specific protein bindings. The membranes were incubated with primary antibodies diluted at a ratio of 1:1000 and left overnight at 4 °C. On the following day, the membranes were washed with 1X TBST three times, 10 min each. After this, the secondary antibodies were added to the membranes for 1 h. For the detection of protein appearance on X-ray, the ECL (EzWestLumiOne, ATTO, Tokyo, Japan) was used on membranes. Finally, the results of protein expression were obtained on X-ray films and it was further analyzed through the computer-based ImageJ software (Version 1.54j).

### 4.8. GSH and MDA Assay

Jawad et al. previously described a procedural approach used to evaluate glutathione reduction, which was followed in this study with a few minor adjustments [63]. The procedure was started by mixing 0.5 mL of freshly prepared DTNB 5,5-dithiobis (2-nitrobenzoic acid) stock solution with 0.1 mL of cortical and hippocampal tissue supernatants in 2.4 mL phosphate-buffered stock solution. After 10 min, the intensity of the resulting yellow color was measured using a spectrophotometer at a wavelength of 412 nm. The obtained GSH concentration values are given in μmol GSH/g of sample. In addition, lipid peroxidation (LPO) rates were calculated using a modified method of Utley et al. by measuring the concentration of malondialdehyde (MDA). The test formulation comprised 200 µL cortical and hippocampal tissue, 20 µL mM ferric chloride, 200 µL 100 mM ascorbic acid, and 580 µL 0.1 mM phosphate buffer (PH 7.4). It was then incubated in a water bath at 37 °C for 60 min. After one hour of incubation, the samples were treated with 1000 μL of 10% trichloroacetic acid (TCA) and 1000 μL of 0.66% thiobarbituric acid (TBA) to stop the reaction. The tubes were kept in the water bath for twenty minutes, cooled in an ice bath, and then centrifuged for ten minutes at 3000× *g*. The concentration of thiobarbituric acid reactive substances (TBARSs) was measured at 535 nm using cortical and hippocampal supernatant absorbance and blank (containing all reagents except test sample). The results were represented as nM TBARS/min/mg protein.

### 4.9. Statistical Analysis

ImageJ software was used to scan and analyze Western blot bands using densitometry. The standard error mean (SEM) was used to express the results. For each experimental group, a one-way analysis of variance (ANOVA) was carried out. Behavioral data comprised 8 mice per group, whereas Western blot and confocal data each consisted of 4 mice per group. The obtained results were suggestive of three different experiments. All experimental data were processed, and graphs were produced using GraphPad Prism (version 8.0, San Diego, CA, USA). *p* values indicating statistical significance were those that were 0.05 or less.

## 5. Conclusions

In conclusion, physcion alleviated LPS-induced neuroinflammation and oxidative stress in adult mice. This protective effect can be attributed to the reduction in neuroinflammation, oxidative stress, and memory impairment. These findings suggest that physcion may possess neuroprotective properties and could be beneficial in treating neurological disorders with a neuroinflammatory origin. However, further studies in other in vivo models and higher mammals are needed to fully understand the molecular mechanisms underlying its therapeutic potential.

## Figures and Tables

**Table 1:** List of antibodies used for Western blot and confocal study.

Antibody	Host	Application	Manufacturer	Catalog Number	Dilution
TLR4	Mouse	WB	Santa Cruz Biotechnology, United States	SC293072	1:1000
Iba-1	Mouse	WB	Santa Cruz Biotechnology, United States	SC39840	1:1000
GFAP	Mouse	WB/IF	Santa Cruz Biotechnology, United States	SC33673	1:1000/1:100
p-NF-кB	Mouse	WB	Santa Cruz Biotechnology, United States	SC136548	1:1000
TNF-α	Mouse	WB/IF	Santa Cruz Biotechnology, United States	SC52746	1:1000/1:100
IL-1β	Mouse	WB	Santa Cruz Biotechnology, United States	SC32294	1:1000
Nrf2	Rabbit	WB/IF	Cell Signaling, United States	12721S	1:1000/100
HO-1	Mouse	WB	Santa Cruz Biotechnology, United States	SC136961	1:1000
PSD-95	Mouse	WB/IF	Santa Cruz Biotechnology, United States	SC71933	1:1000/100
SNAP-23	Mouse	WB	Santa Cruz Biotechnology, United States	SC374215	1:1000

## Data Availability

The authors hereby declare that the data presented in this study will be presented upon request from the corresponding author.
